# Benchmarking Outlier Detection Methods for Detecting IEM Patients in Untargeted Metabolomics Data

**DOI:** 10.3390/metabo13010097

**Published:** 2023-01-07

**Authors:** Michiel Bongaerts, Purva Kulkarni, Alan Zammit, Ramon Bonte, Leo A. J. Kluijtmans, Henk J. Blom, Udo F. H. Engelke, David M. J. Tax, George J. G. Ruijter, Marcel J. T. Reinders

**Affiliations:** 1 Department of Clinical Genetics, University Medical Center Rotterdam, Dr. Molewaterplein 40, 3015 GD Rotterdam, The Netherlands; 2 Department of Human Genetics, Radboud University Medical Center, 6525 GA Nijmegen, The Netherlands; 3 Translational Metabolic Laboratory, Department of Laboratory Medicine, Radboud University Medical Center, 6525 GA Nijmegen, The Netherlands; 4 Center for Molecular and Biomolecular Informatics, Radboud Institute for Molecular Life Sciences, Radboud University Medical Center, 6525 GA Nijmegen, The Netherlands; 5 Faculty of Electrical Engineering, Mathematics and Computer Science, TU Delft, Van Mourik Broekmanweg 6, 2628 XE Delft, The Netherlands

**Keywords:** untargeted metabolomics, outlier detection, anomaly detection, one-class methods, IEM, inborn errors of metabolism

## Abstract

Untargeted metabolomics (UM) is increasingly being deployed as a strategy for screening patients that are suspected of having an inborn error of metabolism (IEM). In this study, we examined the potential of existing outlier detection methods to detect IEM patient profiles. We benchmarked 30 different outlier detection methods when applied to three untargeted metabolomics datasets. Our results show great differences in IEM detection performances across the various methods. The methods *DeepSVDD* and *R-graph* performed most consistently across the three metabolomics datasets. For datasets with a more balanced number of samples-to-features ratio, we found that *AE reconstruction error*, *Mahalanobis* and *PCA reconstruction error* also performed well. Furthermore, we demonstrated the importance of a PCA transform prior to applying an outlier detection method since we observed that this increases the performance of several outlier detection methods. For only one of the three metabolomics datasets, we observed clinically satisfying performances for some outlier detection methods, where we were able to detect 90% of the IEM patient samples while detecting no false positives. These results suggest that outlier detection methods have the potential to aid the clinical investigator in routine screening for IEM using untargeted metabolomics data, but also show that further improvements are needed to ensure clinically satisfying performances.

## 1. Introduction

In recent years, untargeted metabolomics has found its way into the clinic where this platform can be used to screen for inborn errors of metabolism (IEM). It has been shown that this platform can successfully detect a variety of IEM [1,2,3,4,5,6,7,8,9,10,11,12]. Detecting IEM involves the discovery of aberrant patterns of metabolomics profiles and linking them to a certain IEM. However, the interpretation of these profiles is complicated by a growing number of metabolite annotations. Hence, manual analysis of untargeted metabolomics data is time-consuming and as a result currently limited to a set of (annotated) metabolites. When no clear coherent IEM pattern can be found in these metabolites, a decision needs to be made whether to continue with a more in-depth investigation or to stop the investigation without a diagnosis. Yet, potential disease patterns may be found in the unidentified features, but this requires the ability to detect aberrant profiles within the unidentified features.

**Figure 1:**
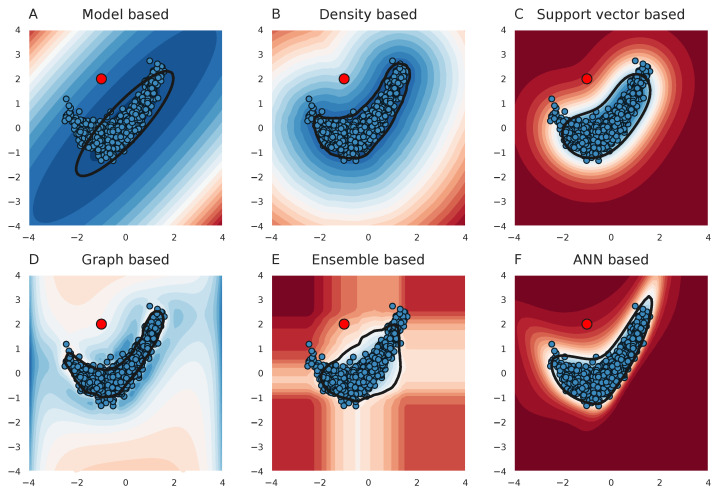
Outlier detection methods try to establish a boundary around the normal samples. Outlier methods are presented with a set of normal samples and extracted from a boundary around them. Distances to this boundary then give an impression of how aberrant a sample is with respect to the set of normal samples. Here, six different outlier methods are shown that are based on distinct approaches. The normal samples on which the outlier methods are trained are presented as blue dots in the plots. The boundary is depicted with the black line and outlier scores (to the boundary) are colored using a heatmap. As an example, one outlier sample is shown, the red dot. The different figures show that different methods use different assumptions with respect to the distribution of the normal samples. As a result, some methods are able to accurately follow the shape of the normal data, whereas other methods find it harder to establish an appropriate boundary. Note that in these illustrations, only two features are considered. (**A**) Model based outlier detection. (**B**) Density based outlier detection. (**C**) Support vector based outlier detection. (**D**) graph based outlier detection. (**E**) Ensemble based outlier detection. (**F**) Artificial neural network (ANN) based outlier detection.

To guide this decision-making process, outlier detection methods can potentially be used to assign an outlier score to each metabolomics profile [13,14]. An increased abnormality, i.e., increased outlier score, could motivate the investigator to continue with a more in-depth investigation for that patient. These methods typically try to establish a boundary such that the majority of the healthy/normal samples lie within this boundary (Figure 1). Outlier samples are considered to be those samples that are located outside this boundary and thus have an abnormal metabolite profile. However, finding such a boundary is not a straightforward task and is complicated by an increasing number of features. It is not surprising that over the course of time, many different machine learning methods have been proposed for the purpose of (generic) outlier detection.

Differences between outlier detection methods can be understood from differences in the assumptions on the distributions of the normal samples, on the shape of the boundary, as well as differences on how to model these distributions or boundaries. A restrictive assumption is to assume that the normal samples are Gaussian distributed. Brini et al. investigated such a methodology, called *ES-CM*, and calculated the Mahalanobis distance for each metabolomics profile. This distance is derived from the (Gaussian) covariance matrix of the normal data [14]. In order to deal with a small number of (normal) samples with respect to the number of features/metabolites, the authors investigated the use of shrinkage estimators to improve the estimate of the covariance matrix. They have shown that IEM patients indeed had higher outlier scores (i.e., Mahalanobis distances) than their normal samples. Although the assumption that the normal data follows a multivariate Gaussian distribution might be beneficial in case a limited number of normal samples is available, this assumption might also lead to reduced performance in IEM detection when the data do not follow this model (e.g., see Figure 1A).

Model-agnostic outlier detection methods could circumvent this issue since they do not rely on any assumption about the shape of the data. For example, non-parametric density-based methods estimate the sample density for a given point in (hyper)space. Outlier samples are positioned in regions with reduced sample density (Figure 1B). Yet, the way in which densities are measured substantially differs from method to method [15,16].

As the objective is to separate the hyper-space into a region containing normal samples versus a region in which there are no normal samples, one can also try to find this decision boundary directly. The one-class support vector machine (*OC-SVM*), is such a method that finds the optimal hyper-plane that separates the normal samples from the origin [17]. With the use of the so-called ‘kernel trick’, more tight non-linear boundaries can be established. Similarly, Tax et al. developed a support vector data description (*SVDD*), that uses the same mathematical principles as *OC-SVM* but defined a (mathematical) problem that solves for a hypersphere with minimal volume that contains the majority of the normal data [18].

Similarity between the normal samples can also be expressed by creating a graph representation of the normal samples. For example, samples (nodes) are connected (edge) when the distance between them in the feature space is small [19], or by describing each sample as a linear combination of other samples [20]. Hence, the graph describes the local topology of similar normal samples. The obtained graph could then be used to calculate the outlier scores. For example, *R-graph* propagates scores through the graph using a Markov process to calculate an outlier score per sample. In this case, it is expected that the score is lower for an outlier sample since more ‘score’ flows away from the (outlier) sample to other samples than is received by the outlier sample from its neighbors.

Instead of relying on one outlier detection method, one can also use the agreement between multiple outlier detection methods. Ensemble methods combine the results from many individual (simple) outlier detectors in order to improve performance. For example, *Isolation Forest* uses random splits in random features to segregate samples [21]. For an outlier sample, it is expected that on average lesser splits are needed to isolate that sample.

More recently, methods based on artificial neural network (ANN) architectures have been proposed for the purpose of outlier detection. These methods have been mostly applied to image datasets in order to detect abnormal images or abnormalities in images. Oza et al. proposed a method, called *OC-CNN*, where a classifier network is trained to distinguish artificial noise (i.e., outliers) from normal samples. Informative features were first obtained for each sample using a ‘feature extractor’ before it was used as input for the classifier [22]. Based on *SVDD*, the *DeepSVDD* method uses an ANN in order to perform the required non-linear mapping [23].

Other ANN methods integrated the generative adversarial network (GAN) architecture to perform outlier detection [24,25,26,27]. A GAN consists of a generator and a discriminator network, where the generator has the task of generating artificial samples that closely resemble the normal samples, while the discriminator tries to discriminate between artificial and normal samples. The key idea is to use the discriminative power of the discriminator as a loss for the generator during training [28]. Several methods have been proposed to perform outlier detection using GANs but they differ in network architecture and the way outlier scores are acquired from the (trained) network.

Outlier detection performed on metabolomics data with the purpose of detecting IEM patients has been reported previously in two studies [13,14]. Both studies showed that IEM patients have increased outlier scores when using the outlier detection method as proposed by these authors. Nevertheless, both studies investigated the use of a single type of outlier detection method and applied that method to a single (IEM) dataset. To our knowledge, no study has been reported that explored a large set of diverse outlier detection methods and applied those methods to several metabolomics datasets.

Although not for metabolomics data, outlier detection methods have been benchmarked for a variety of different types of data. For example, Han et al. showed that none of the 14 investigated outlier detection methods were significantly better when compared to each other and applied to 57 different problems [29]. On the contrary, Campos et al. found significant performance differences when comparing 12 distinct different outlier detection methods to 23 distinct datasets [30]. Generally, it seems that the performance of each outlier detection method largely depends on the dataset to which it has been applied. Additionally, the majority of outlier detection methods contain (hyper)parameters that require ‘tuning’ and not all studies tackled this issue in the same way, which, therefore, might also lead to varying outcomes.

We set out to compare 30 different outlier detection methods specifically to detect IEM patients from untargeted metabolomics data. The majority of these 30 methods originated from the open-source libraries Scikit-learn [31] and PyOD [32], whereas the remaining methods were obtained from individual studies and/or manually implemented for this study. We evaluated these outlier detection methods on three independent untargeted metabolomics datasets. Our results suggest that certain methods are evidently more suitable for the purpose of detecting IEM patients as compared to others. Moreover, state-of-the-art methods did not necessarily result in improved performance when compared with the more conventional methods.

## 2. Materials and Methods

### 2.1. Evaluating the Performance of Each Outlier Detection Method

In order to evaluate the performance of each outlier detection method on the detection of IEM patients, we calculated the area under the curve (AUC) of the receiver operating characteristic (ROC). This curve is created by displaying the fraction of IEM patients having an ‘outlier score’ above a given cut-off value as a function of the fraction of normal samples (from the evaluation/test set) having a score above that same cut-off. The area under the ROC curve expresses the overall detection performance of a method, where an AUC closer to 1 indicates improved performance.

Furthermore, we choose to evaluate two points at the ROC curve that we considered clinically interesting: (1) the point closest to the (0, 1) point and (2) the point at which 90% of the IEM patient samples are labeled as ‘outlier’. At these points, we computed the balanced accuracy, precision, and recall for both the IEM patient and normal samples. These metrics are given by the following equations:

(1) $${\mathrm{Precision}}_{P}=\frac{\mathrm{TP}}{\mathrm{TP}+\mathrm{FP}}$$

(2) $${\mathrm{Precision}}_{N}=\frac{\mathrm{TN}}{\mathrm{TN}+\mathrm{FN}}$$

(3) $${\mathrm{Recall}}_{P}=\frac{\mathrm{TP}}{\mathrm{TP}+\mathrm{FN}}$$

(4) $${\mathrm{Recall}}_{N}=\frac{\mathrm{TN}}{\mathrm{TN}+\mathrm{FP}}$$

(5) $$\mathrm{Balanced}\;\mathrm{accuracy}=\frac{1}{2}\left(\frac{\mathrm{TP}}{P}+\frac{\mathrm{TN}}{N}\right)=\frac{1}{2}\left({\mathrm{Recall}}_{P}+{\mathrm{Recall}}_{N}\right)$$

### 2.2. Cross-Validation and Parameter Selection

The majority of outlier detection methods have (hyper)parameters that require ‘tuning’. Furthermore, when evaluating the performance of the various methods on each dataset, we need to use a cross-validation procedure where training samples are used to train the detector and where a test set is used to build the ROC curve. In this study, we chose to perform cross-validation using an evaluation set to decide which settings for the (hyper)parameters were optimal, and cross-validation using a test set to evaluate the performance of each method on IEM detection. Since the number of available normal samples for the Miller and Radboudumc dataset was relatively low, we decided to perform these cross-validation procedures in a slightly different manner than the Erasmus MC dataset. These procedures are described in this section.

#### 2.2.1. Erasmus MC Dataset

The Erasmus MC dataset consisted of 112 IEM patient samples, 10 samples with an abnormal metabolite profile, and 522 patient samples without IEM-related diagnosis (see Appendix A for more details). The latter group was assumed to be a normal/reference cohort. Six cross-validation datasets were created by randomly selecting 70 (normal) samples for the evaluation/test set. The known IEM patient samples were always included in the test/evaluation set and thus excluded from the train set. For each cross-validation experiment (CV), an ROC curve was created using the outlier scores from the IEM patients and the normal samples were selected from that CV. Three out of the six CVs were used to evaluate which parameter settings were best by calculating the mean AUC from these three CVs (i.e., evaluation set). Next, the final average AUC was taken from the remaining three CVs (i.e., test set).

#### 2.2.2. Miller Dataset

The Miller dataset consists of 120 known IEM patient samples and 70 normal samples (see Appendix A for more details). We used 18 cross-validation experiments (CV), each having four normal samples for the test/evaluation set; the remaining 66 normal samples comprised the train set. Outlier scores for the normal samples from 9 out of the 18 CVs were pooled together and formed the evaluation set. Similarly, the outlier scores from the remaining nine CVs were also pooled together to form the test set. For each cross-validation experiment, two ROC curves were created using the outlier scores from the IEM patients as determined from that CV and by bootstrapping the (pooled) outlier scores from the normal samples from the evaluation set and the test set. In other words, 18 bootstrapped ROC curves were obtained from the (pooled) evaluation set, and 18 curves from the (pooled) test set. The optimal (hyper)parameter settings were chosen from the highest average AUC calculated from the 18 evaluation AUCs. The 18 test AUCs were used to calculate the final average AUC.

#### 2.2.3. Radboudumc Dataset

The Radboudumc dataset consists of 38 known IEM patient samples, three samples with an abnormal metabolite profile, and 123 normal samples (see Appendix A for more details). We used seven cross-validation experiments, each having 18 normal samples for the evaluation/test set, except for one CV having 15 normal samples. Similar to the analysis described in Section *Evaluation and parameter selection Miller dataset*, we pooled the outlier scores for the normal sample for three out of the seven CVs for the evaluation set. The outlier scores for the normal samples in the remaining four CVs were pooled together to comprise the test set. For each cross-validation experiment, two ROC curves were created using the outlier scores from the (true) outlier samples, as determined from the CV and bootstrapping the (pooled) outlier scores from the normal samples from the evaluation set and the test set. The optimal (hyper)parameter settings were chosen from the highest average AUC calculated from the seven evaluation AUCs. The seven test AUCs were used to calculate the final average AUC.

## 3. Results

We compared 30 different outlier detection methods on three different datasets. The characteristics of the three datasets are summarized in Table 1 and details are given in Appendix A. The three metabolomics datasets differ in the number of features, number of normal and IEM patient samples, and number of distinct IEM included. Note that each metabolomics dataset was acquired from a different experimental set-up and varied in data (pre-)processing, i.e., peak alignment, peak peaking, peak integration, normalization etc. [33]. This variety is favorable since it allows us to study the consistency of each outlier detection method on IEM detection across different datasets.

For all datasets, normal samples and abnormal/patient samples were available. The outlier detection methods were trained only on normal samples and evaluated using a cross-validation procedure (see *Methods*). Briefly, the outlier detection methods were evaluated on how well they can separate known normal and known abnormal samples (i.e., IEM patient samples) that were not seen during training. Performance was expressed in both the area under the receiver operating characteristic (ROC) curve (AUC), as well as two clinically relevant points at the ROC curve: (1) the point for which the performance of the outlier detector is closest to the optimal performance (detecting all patients (true positives), while not calling any of the normal samples (false positives)) and (2) the point at which 90% of the known IEM patients were detected (true positive rate or recall${}_{P}$ equal to 0.9), assuming that this is a satisfying detection rate for the clinic. At both points, we determined the balanced accuracy, recall, and precision for each method (see *Methods*).

### 3.1. Performance Differences across Methods

**Figure 2:**
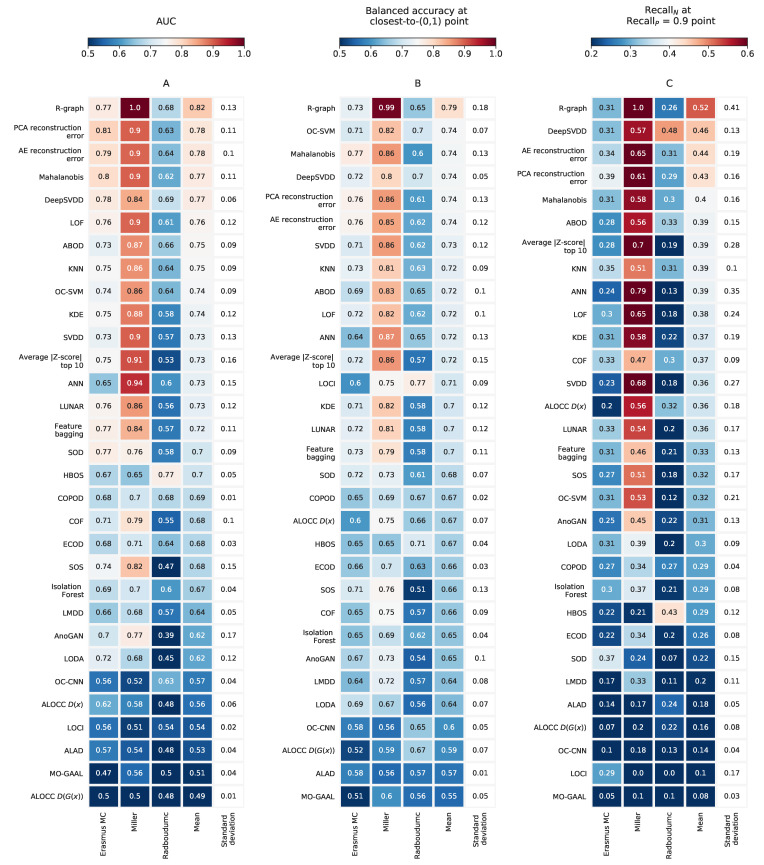
Several performance metrics are used to evaluate the various outlier detection methods. (**A**) Average (across the cross-validations) AUC of the ROC curves for each dataset and outlier detection method. In addition, the average and standard deviation of the AUC across all three datasets are reported. The methods are sorted based on this average AUC. (**B**) Average balanced accuracy (see Methods) at the ’closest-to-(0,1)’ point of the ROC curve for each dataset and outlier detection method. Again, the methods are sorted based on the average balanced accuracy across the three datasets. (**C**) Recall${}_{N}$ at the ‘Recall${}_{P}$ = 0.9’ point at the ROC curve.

Figure 2A shows the average AUC for each investigated outlier detection method and dataset. We were interested in those methods that perform well regardless of the differences between datasets. By sorting the methods based on the average AUC across the three datasets (as in Figure 2A), we observe that *R-graph* and *DeepSVDD* had a (relatively) good and consistent performance across datasets. It is worth noting that the standard deviation on the AUC for *DeepSVDD* applied to the Radboudumc was relatively high (Appendix L), indicating that the performance was not consistent across the different train and test sets. The *ANN* method had a high performance for the Miller dataset but performed less on the Erasmus MC and Radboudumc dataset. When maximizing the performance per dataset, we observe that *PCA reconstruction error* was optimal for the Erasmus MC dataset (AUC = 0.81), *R-graph* is optimal for the Miller dataset (AUC = 1) and *HBOS* is optimal for the Radboudumc dataset (AUC = 0.77). Note that *HBOS* performed poorly on the Erasmus MC and Miller dataset.

We observe that reconstruction-based techniques, i.e., *PCA reconstruction error* and *AE reconstruction error* performed relatively well on the Erasmus MC and Miller dataset but poorly on the Radboudumc dataset. The same holds for the *Mahalanobis* method. Since the dimensionality (i.e., number of features) with respect to the number of normal samples was much larger for the Radboudumc dataset than for the other two datasets (see Table 1), we assume that *PCA reconstruction error*, *Mahalanobis,* and *AE reconstruction error* are more sensitive to the number of normal samples in the train set.

Poor performing methods were *ALOCC*, *ALAD*, *COPOD*, *ECOD*, *Isolation Forest*, *LOCI*, *LMDD*, *MO-GAAL,* and *OC-CNN* having an AUC of ≤0.71 for all datasets. This indicates that investigators may want to avoid these methods for the purpose of detecting IEM patients. Yet, when we reduced the dimensionality by performing principle components analysis (PCA)—applied on all samples in the dataset (including the train and test set)—we were able to increase the AUC for several poor-performing methods when applied to the Erasmus MC dataset (see Appendix D). For example, using 60 principle components (PCs), the AUC for *Isolation Forest* went from 0.69 to 0.78. Similarly, the AUC for *ECOD* and *COPOD* went from 0.68 to 0.78 using 150 and 60 PCs, respectively. *LMDD* improved from 0.66 to 0.76 using 150 PCs. These results show that performing PCA prior to applying an outlier detection method may be beneficial for a subset of methods. Yet, none of these approaches performed better than *PCA reconstruction error* without the initial PCA step. Additionally, we performed a similar experiment on the Radboudumc dataset for a subset of outlier detection methods (see Appendix E). These results confirm that an initial PCA transform could improve performances. For *PCA reconstruction error* using 20 PCs, we were able to obtain an AUC of 0.84 for this dataset. Using 20 PCs, *Mahalanobis* and *LOF* obtained an AUC of 0.75 and 0.82, respectively, which is a clear improvement over the situation where the PCA transform has not been applied (i.e., Figure 2A).

In this study, we observed some complications with the training of the GAN-based methods which may, at least partially, explain their poor performance. *ALOCC* training on the Erasmus MC dataset resulted in an increasing loss for the generator, indicating that the discriminator was always winning from the generator (see Appendix H). One-sided label smoothing was supposed to prevent this type of behavior, but was unsuccessful [34]. The authors of *ALOCC* proposed to stop the training when the reconstruction loss achieved a certain value. However, training *ALOCC* on the Erasmus MC dataset did not result in a decreasing reconstruction loss either, which complicated the use of this stopping criterion. Training *ALOCC* on the Miller dataset did result in a decreasing generator and reconstruction error, but its performance on IEM detection was still among the worst. Ever-increasing generator loss and decreasing discriminator loss were also observed for *AnoGAN*, *ALAD,* and *MO-GAAL* (see Appendix I, Appendix J and Appendix K). GAN-based methods furthermore involve the training of many parameters (typically in the order of millions), which makes training computationally expensive. Altogether, these observations show that training GAN-based outlier detection methods is not a straightforward task.

### 3.2. Performance Differences across Datasets

**Figure 3:**
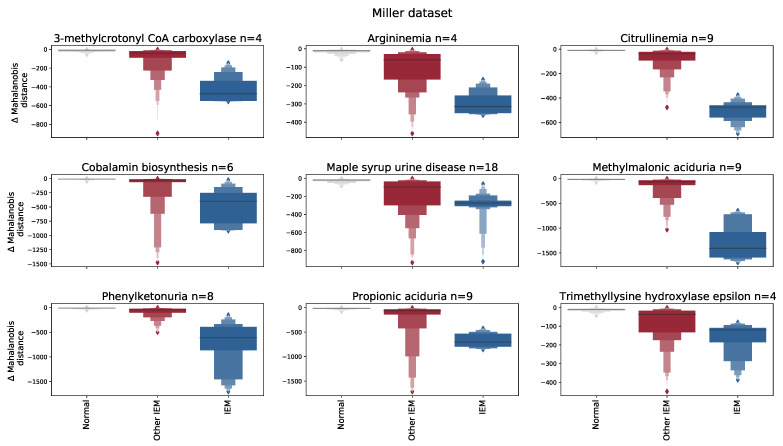
Each panel shows the difference (Δ) in Mahalanobis distance between the situation that all 52 biomarkers (as stated in the study of Miller et al.) were included and the situation that IEM-related biomarkers were removed from the dataset. Note that for each IEM (i.e., panel) a different set of biomarkers is left out. A negative difference in Mahalanobis distance indicates that the Mahalanobis distance decreased (i.e., reduced abnormality) when IEM-related biomarkers are removed with respect to the situation that all 52 biomarkers are included. These differences are shown for three groups: normal (test) samples (grey), IEM patient samples having an IEM other than the IEM stated in the title (red), and IEM patients having the IEM as stated in the title (blue). The ‘n’ in the title indicates the number of patient samples that are included with that particular IEM.

Figure 2A also shows that AUCs vary across the explored datasets, with overall higher performances for the Miller dataset. We expect that this is mainly a consequence of the fact that the Miller dataset contains only 26 distinct IEM and contains biomarkers for each IEM, thereby easing the task of detecting the IEM patients as outliers. In order to support this argument, we compared the Mahalanobis distance of the IEM patient samples with and without the inclusion of these IEM-related biomarkers (see Figure 3). From this experiment, we clearly observe a decline in the Mahalanobis distance(s) for the IEM patient samples when the relevant biomarkers were removed from the dataset.

The highest AUC achieved for the Erasmus MC dataset was 0.81. Since 62 distinct IEM are included in this dataset, we expect that detecting all these distinct IEM might be a more challenging task. Furthermore, the Erasmus MC dataset has the lowest number of features due to the specific pre-processing steps that were followed. Consequently, several IEM-related features may have been absent from this dataset which might have considerably reduced the ability to detect IEM patient samples.

The majority of methods performed poorly on the Radboudumc dataset—only three methods performed relatively well (e.g., AUC ≥ 0.68). The poor performance of the majority of the outlier detection methods we relate to the small number of available normal samples for training with respect to the number of features. Indeed, we saw that reducing the dimensionality (using a PCA step) positively affects the performance of a number of poorly performing methods. This suggests that for new datasets, it is important to explore the dimensionality reduction before applying the outlier detection methods.

### 3.3. Clinical Relevance of Outlier Detection Methods on Detecting IEM Patients

When evaluating the outlier detection methods for their optimal performance (i.e., the performance on the ROC curve closest to the (0,1) point), we found that for the three metabolomics datasets, *R-graph* had recall rates for the IEM patients (recall${}_{P}$) ranging between 0.62–0.98 (see Appendix F). Recall rates for the normal samples (recall${}_{N}$) were in the range of 0.69–1.00. Similarly, for *DeepSVDD* recall${}_{P}$ ranged from 0.72 to 0.81 and recall${}_{N}$ ranged from 0.62 to 0.8. When maximizing the balanced accuracy per dataset and method, we observe that for the Erasmus MC dataset, *Mahalanobis* had a balanced accuracy of 0.77 (Figure 2B) with recall${}_{P}$ = 0.7 and recall${}_{N}$ = 0.85. For the Miller dataset, *R-graph* had a balanced accuracy of 0.99 with recall${}_{P}$ = 0.98 and recall${}_{N}$ =1.00. For the Radboudumc dataset, *HBOS* had a balanced accuracy of 0.71 with recall${}_{P}$ = 0.68 and recall${}_{N}$ = 0.75.

When looking at the ‘recall${}_{P}$ = 0.9’ point, we observe that for the Erasmus MC dataset and *PCA reconstruction error,* the recall${}_{N}$ = 0.39, indicating that 61% of the normal samples were false positives (see Figure 2C). For the Miller dataset and *R-graph,* this recall rate was 1, which suggests clinically satisfying performances. *HBOS* applied on the Radboudumc dataset had a recall${}_{N}$ of 0.43. Altogether, this shows that for high IEM recall rates (i.e., recall${}_{P}$ = 0.9), we should also expect a significant percentage (0–61%) of false positives. As described above, we were able to obtain an AUC of 0.84 by performing an initial PCA transform on the Radboudumc dataset and using *PCA reconstruction error*. In this case, for the ‘recall${}_{P}$ = 0.9’ point, we observe that *PCA reconstruction error* had a recall${}_{N}$ = 0.57 (see Appendix E).

## 4. Discussion

The aim of our study was to investigate the potential of outlier detection methods to detect IEM patients as outliers in untargeted metabolomics data. Our results show that *DeepSVDD* and *R-graph* are two methods that performed consistently well across the three datasets when looking at the AUC. The methods *AE reconstruction error*, *Mahalanobis,* and *PCA reconstruction error* were effective for detecting IEM patients for the Erasmus MC and Miller dataset, thereby partially confirming the results previously obtained by Brini et al. [14] and Engel et al. [13]. When maximizing the AUC for each dataset individually, we observed that *PCA reconstruction error* was optimal for the Erasmus MC dataset, *R-graph* was optimal for the Miller dataset, and *HBOS* was optimal for the Radboudumc dataset. These findings support results from previous studies that show that the best-performing method largely depends on the dataset on which it is applied to [29,30].

Evidently, we have seen that a subset of outlier detection methods has predictive power to detect IEM patients in metabolomics data (e.g., AUC ≫ 0.5). However, in order to judge whether such a strategy could successfully be used in the clinic we evaluated the methods also on their performance when 90% of the IEM patients were detected. Given this requirement, we have seen that *PCA+PCA reconstruction error,* (i.e., *PCA reconstruction error* with an initial dimensionality reduction by PCA) had the best performance on the Radboudumc dataset with 43% false positives, i.e., normal samples called to be IEM patients. For the Miller dataset, *R-graph* had no false positives for this operating point. However, for the Erasmus MC dataset, the best method was *PCA reconstruction error,* which generates 61% false positives in this clinical setting. This poor(er) IEM detection performance in the Erasmus MC dataset might be related to the relatively high number of distinct IEM and the possible absence of relevant biomarkers in this dataset. This absence of biomarkers is likely to be caused by the fact that only features were included that were measured/detected in at least 20 out of the 25 batches. This relatively strict criterion might have led to the exclusion of IEM-related features and therefore may partially explain the reduced IEM detection performances (i.e., AUC) of the outlier detection methods for this dataset.

We showed that the use of an initial PCA transform could improve the IEM detection performance of several outlier detection methods. However, we need to realize that for this PCA step, we used the full dataset, i.e., the training set (consisting of normal samples) as well as the test set (consisting of normal as well as IEM samples). Therefore, it is expected that the resulting reduced PCA space indirectly acquired information about the IEM patient samples, i.e., information on where IEM samples are distributed with respect to the normal samples is provided. Although the outlier detection methods were trained solely on the training set, the reduced PCA space might represent a subspace in which the normal and IEM samples can be better separated, thereby making it easier for the outlier detection method to find an appropriate boundary. Yet, this is still a valid procedure in the clinical setting when samples are acquired batch by batch. Namely, the proposed procedure (i.e., PCA dimension reduction based on train and test samples, followed by training an outlier detection on the train samples) can similarly be adopted in the clinical setting. Based on the newly acquired batch of samples together with the available training set of normal samples, we can apply the PCA dimension reduction, and after that, train the outlier detection method in this new PCA subspace on the training samples. As we emulated this setting during validation, our reported performance measures will be accurate estimates of the performance in this clinical setting. We stress that it is important that in this setting one needs to redo the PCA dimension reduction using the newly acquired batch of samples, as well as retrain the outlier detector, for every new batch of samples.

Our results suggest that the optimal outlier detection method differed per dataset. Ideally, an investigator would like to know a priori which method should be used given, for example, a certain experimental set-up. The limited number of included datasets (*n* = 3) in this study was not sufficient to study how the differences between these datasets affect the selection of an optimal outlier detection method. A large number of diverse datasets would be needed in order to study this effect and is hampered by the limited availability of untargeted metabolomics studies that study IEM.

All three datasets were Z-score-scaled prior to training the outlier detection methods (see Appendix A). In this study, we performed two distinct methods for scaling. For the Erasmus MC and Radboudumc dataset, the mean and standard deviation per feature were obtained in a robust manner using an iterative procedure where outlier samples were removed. Here, it is assumed that the majority of the samples are normal when considering a single feature. The Miller dataset was scaled based on the control group. Interestingly, when we re-scaled the Miller dataset similarly to the Erasmus MC and Radboudumc dataset, we observed a performance drop for the majority of investigated outlier detection methods (see Appendix M), implicating that further investigation on scaling and appropriate reference sets is important.

Additionally, the normal samples used in the Erasmus MC, Miller, and Radboudumc datasets were acquired from routine screening. Thus, normal samples were assumed to be those samples that did not receive a diagnosis related to a metabolomics disorder. Consequently, reported IEM detection performances in this study may have been biased by the absence of a genuine healthy population.

Besides the differences in the number of distinct IEM, number of normal samples, number of IEM patient samples, and the number of features, we speculate that at least two other factors might also contribute to the IEM detection performance differences between the datasets. Firstly, technical variation (e.g., between experimental batches) in the data may obscure/dilute structures in the data that would normally benefit outlier detection. Adequate removal of these variations (i.e., normalization) is therefore important, and the precision at which this has been achieved might differ between the studied datasets. Secondly, other pre-processing steps, e.g., peak integration, scaling, and data transformation could contribute to differences across the datasets.

Various outlier detection methods contain one or more hyperparameters that ideally need tuning. In this study, we used a parameter sweep for some of these parameters to at least partially ‘tune’ these settings. Especially for methods that use an ANN, many hyperparameters are present (such as the number of hidden layers, number of nodes, type of activation, learning rate, etc.), thereby making a parameter sweep over all parameters computationally unfeasible. Some parameters were chosen to be fixed or were made dependent on the dimension of the input (i.e., *M*) or number of samples in the train set (i.e., *N*) (see Appendix B). We acknowledge that the range of settings that were explored per method was limited and that the ‘true’ optimal setting for a given method might have been in an unexplored subset of settings.

## 5. Conclusions

We have shown that several outlier detection methods have the ability to detect IEM patients in (untargeted) metabolomics data. From the 30 explored outlier detection methods, such as *AE reconstruction error*, *DeepSVDD*, *Mahalanobis*, *PCA reconstruction error,* and *R-graph,* seemed to perform overall best across the investigated metabolomics datasets. The state-of-the-art methods (such as GAN-based methods) did not necessarily outperform the more conventional approaches. Additionally, we showed that performing a PCA transformation prior to applying an outlier detection method generally improves the performance for a subset of methods. Although some methods seem more suitable for the purpose of detecting IEM patients in metabolomics data, our results demonstrate that in the end, the best-performing outlier detection method depends on the dataset to which it is applied.

For only one of the three metabolomics datasets were we able to demonstrate clinically satisfying true and false positives rates, where 90% of the IEM patient samples can be detected while marking none of the normal samples as outliers (i.e., false positives). At this point, for the other two datasets, the (lowest) false positive rates were 43% and 61%, indicating that outlier detection methods may not have clinically satisfying performances. Although we demonstrated that several outlier detection methods have the ability to detect IEM patient samples in metabolomics data, we anticipate that future successes largely depend on the number of distinct IEM that are deemed to be detected, the requirement that IEM-related features are included, and the presence of a genuine normal reference set. In case these requirements are met, we believe that outlier detection could be a useful additional tool in the clinic.

## Figures and Tables

**Table 1:** Overview of the datasets used in this study and their characteristics. See Appendix A for more details about each dataset. * All samples are from a single experimental batch measured across three different set-ups. ** The authors only indicate that the majority of the patients received treatment. ^†^ Only annotated features for this dataset were used in this study.

Dataset	Experimental Set-Up	Tissue Type	# Experimental Batches	# Normal Samples	# Abnormal Samples	# Different IEM	Receiving Treatment	# Features
Erasmuc MC [3]	LC-MS(+)	Blood plasma	25	552	122	62	50%	307
Miller et al. [1]	GS-MS & LC-MS(+/−)	Blood plasma	1 *	70	120	21	>50% **	661 ^†^
Radboudumc [2]	LC-MS(+)	Blood plasma	12	123	41	28	≈75%	6362

**Figure A1:**
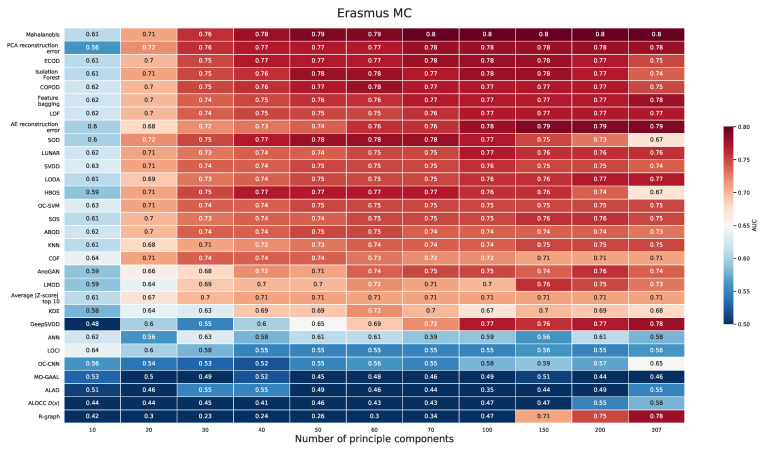
Average AUC (across 4 CVs) for an increasing number of principle components after performing PCA analysis. Hyperparameter settings were fixed for each method (see Table A3).

**Figure A2:**
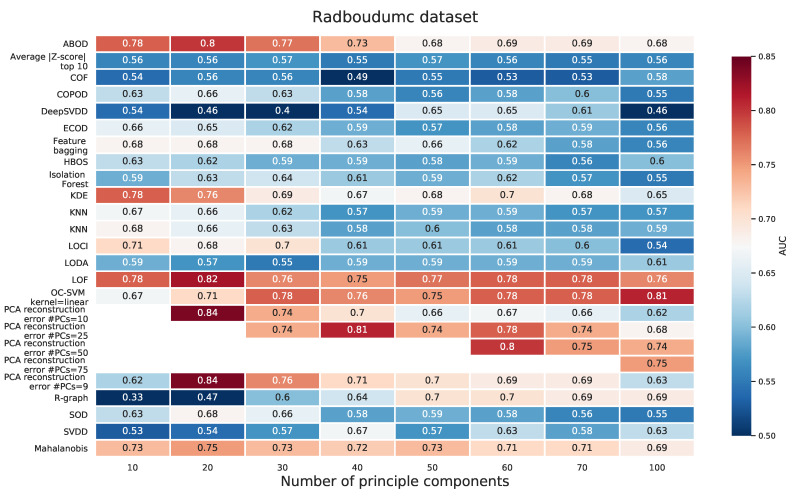
Average AUC (across 7 test CVs) for an increasing number of principle components after performing PCA analysis, similar to as described in Methods. Note that the number of PCs that *PCA reconstruction error* uses should always be lower or equal to the number of PCs that is used to (PCA) transform the dataset.

**Figure A3:**
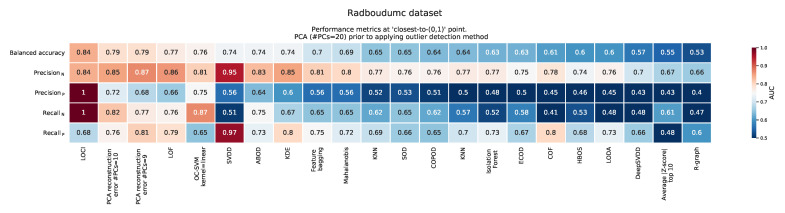
Evaluation metrics at the ‘closest-to-(0,1)’ point for four methods as indicated on the x-axis when 20 PCs are used to transform the dataset.

**Figure A4:**
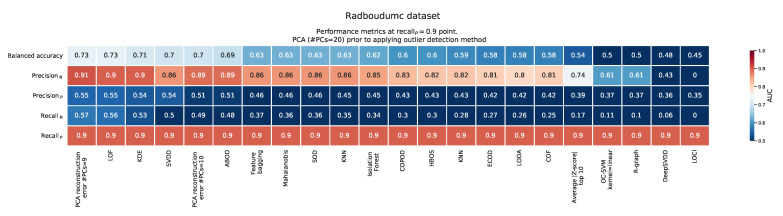
Evaluation metrics at the ‘recall${}_{P}$=0.9’ point for four methods as indicated on the x-axis when 20 PCs are used to transform the dataset.

**Figure A5:**
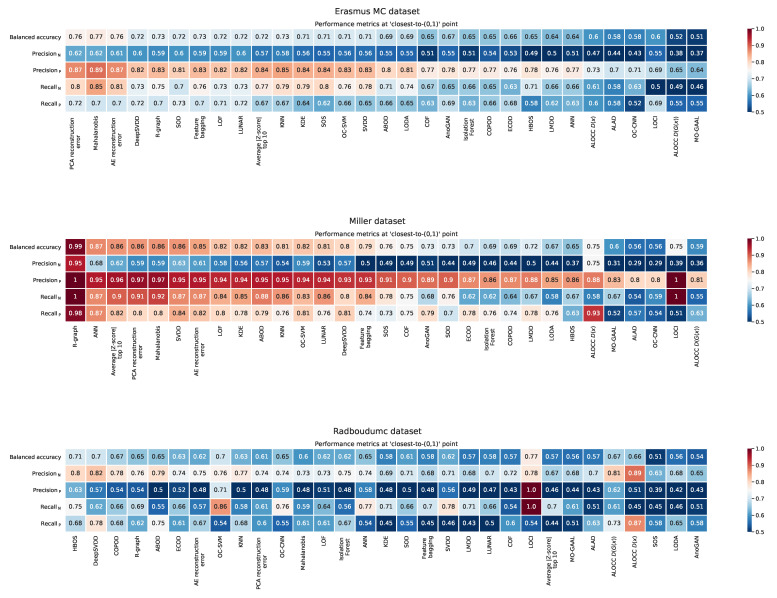
(Average) balanced accuracy, recall, and precision at the point in the ROC curve that lies closest to the (0,1) point. Methods are ordered by descending average AUC per dataset. All reported values are the average across all (test) cross-validations.

**Figure A6:**
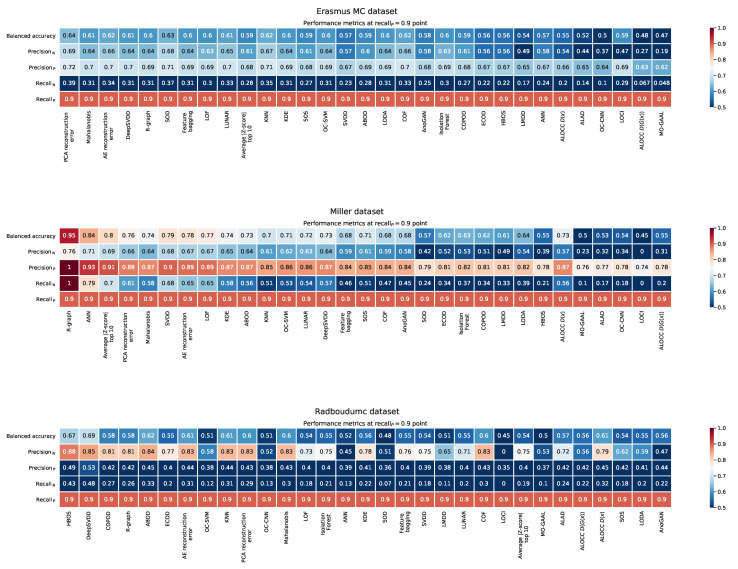
Balanced accuracy, recall, and precision at the point in the ROC curve where recall${}_{P}$ = 0.9. All reported values are the average across all (test) cross-validations.

**Figure A7:**
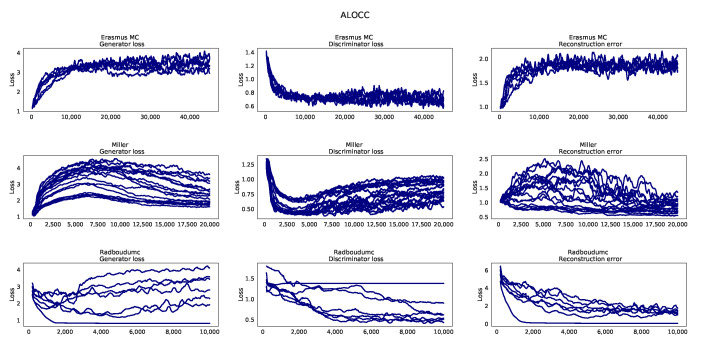
Learning curves for the *ALOCC* method. The generator, discriminator, and reconstruction loss are displayed in each subplot. Note that each curve represent one cross-validation.

**Figure A8:**
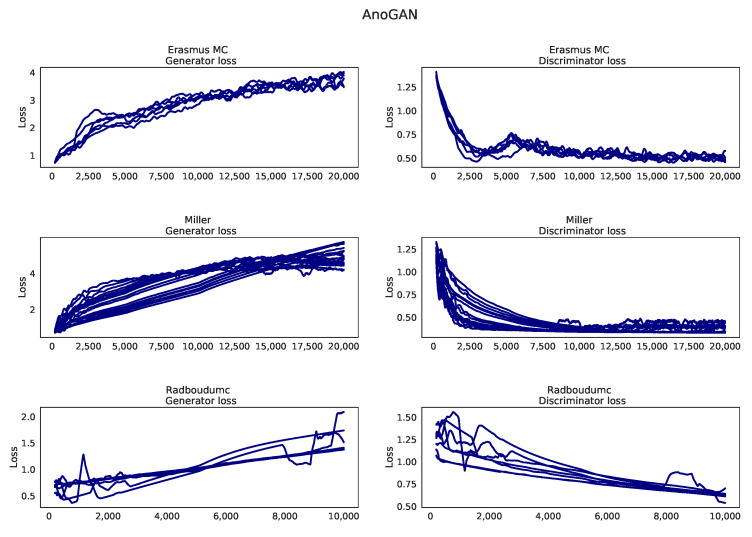
Learning curves for the *AnoGAN* method. The generator and discriminator loss are displayed in each subplot. Note that each curve represent one cross-validation.

**Figure A9:**
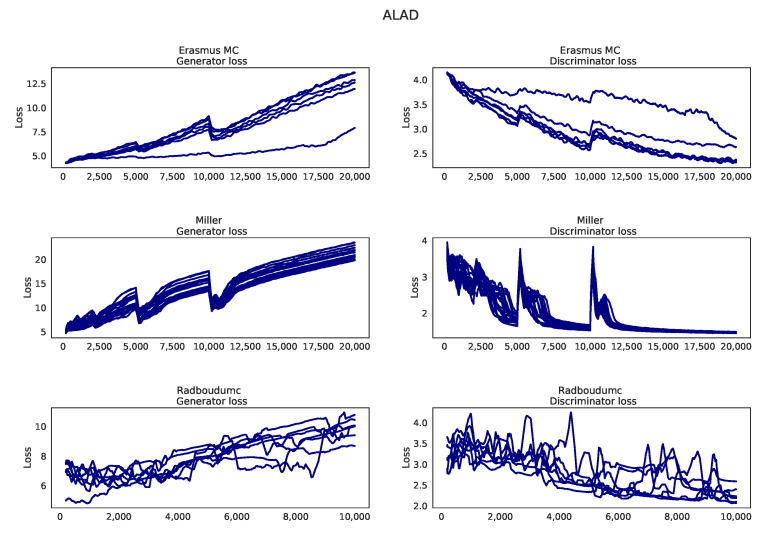
Learning curves for the *ALAD* method. The generator and discriminator loss are displayed in each subplot. Note that each curve represents one cross-validation.

**Figure A10:**
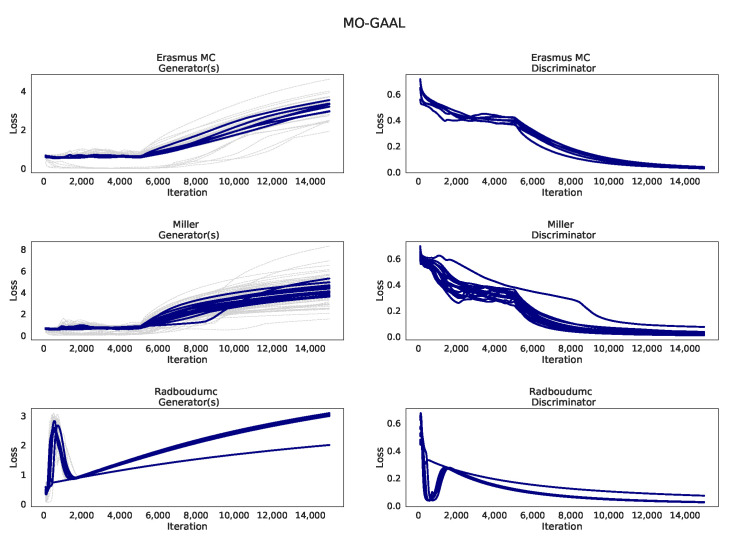
Learning curves for the *MO-GAAL* method with k = 5 (number of subgenerators). The generator and discriminator loss are displayed in each subplot. Note that each curve represent one cross-validation. The grey learning curves indicate the individual losses of the subgenerators.

**Figure A11:**
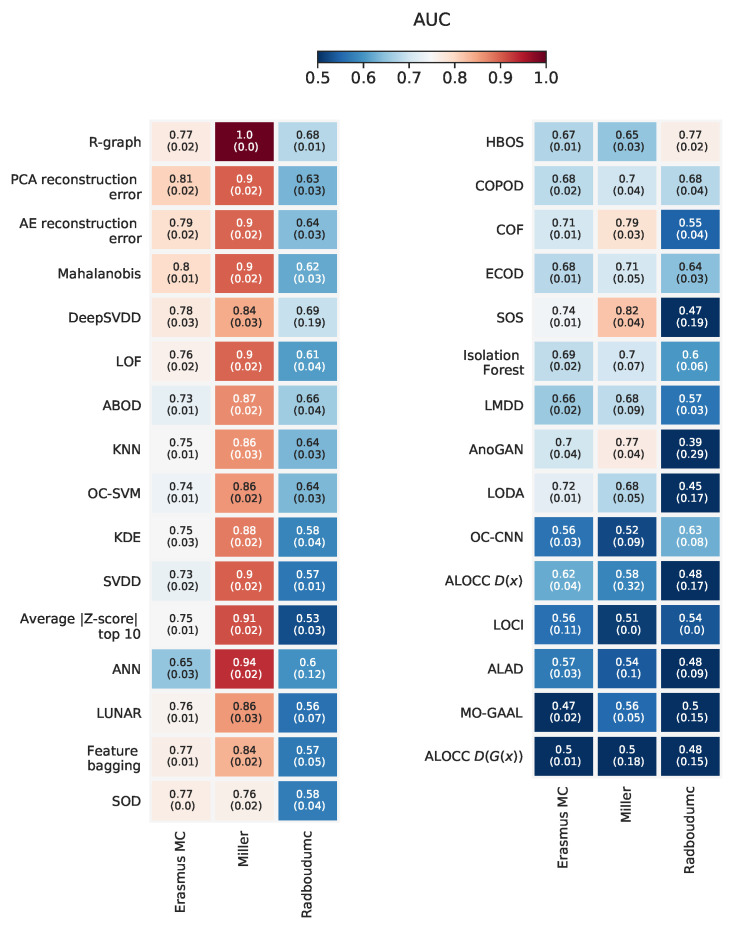
Overview of the average AUC plus standard deviation (between parentheses) on the AUC obtained from the k-fold cross-validation.

**Figure A12:**
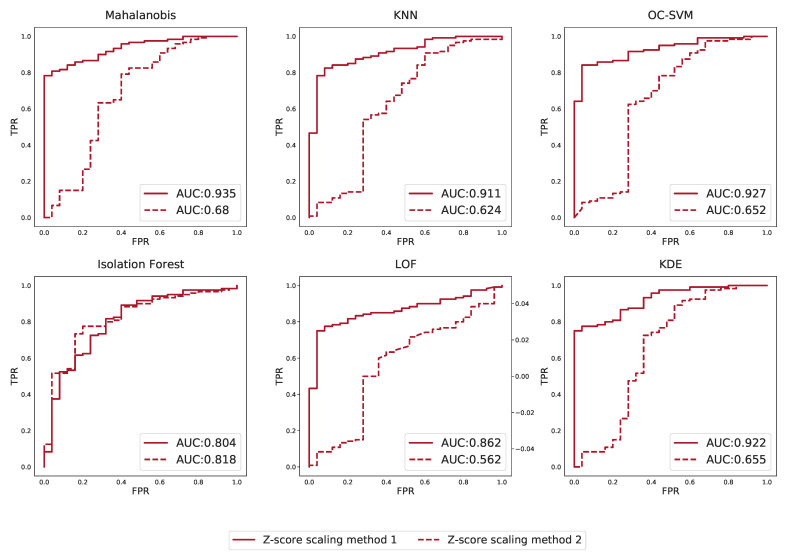
ROC curves and AUCs for six outlier detection methods (as indicated in the title), for two different Z-score scaling methods.

## Data Availability

Not applicable.

## Abbreviations

    The following abbreviations are used in this manuscript:

ANN	Artificial neural network
AUC	Area under the curve
CV	Cross-validation
GAN	Generative adversarial network
ROC	Receiver operating characteristic
IEM	Inborn error of metabolism
PCA	Principle component analysis
PC	Principle component
UHPLC	Ultra-high performance liquid chromatography

## Appendix A. Datasets

### Appendix A.1. Erasmus MC Dataset

Untargeted metabolomics data from the blood plasma of 674 unique samples was measured in 25 separate batches [3] and was merged to form a single dataset. This was done by matching features from one batch to a reference batch (that was chronologically in the middle) [35]. A feature was included in the merged dataset only if it was matched/detected in at least 20 other batches. For the remaining missing values, an autoencoder was used to impute these missing values (see *Autoencoder*). Furthermore, no significant differences were present in the number of missing values between the IEM patient group and the normal group. This was accessed by determining the number of missing values per sample within each group and applying the Mann–Whitney U test. For the six cross-validations (see *Evaluation and parameter selection Erasmus MC dataset*), the resulting *p*-values were in the range of 0.12–45. The final dataset contains 307 features, where 98 features received a metabolite identification. We used *Metchalizer* with a Box–Cox transformation to correct for batch effects and other technical drifts [35]. Although each batch was measured in both negative and positive ion mode, we only included the positive ion mode for this study.

The dataset contains 552 unique samples obtained from patients that did not receive a diagnosis related to a metabolic disorder. Further, 112 patient samples were included with known IEM and 10 samples with an abnormal metabolomics profile not related to an IEM. The following IEM were included: Medium chain acyl-CoA dehydrogenase deficiency (*n* = 6), carnitine palmitoyltransferase II (*n* = 5), nonketotic hyperglycinemia (*n* = 5), phenylketonuria (*n* = 5), methylmalonic acidemia (*n* = 4), homocystinuria (*n* = 4), isovaleric acidemia (*n* = 3), Smith–Lemli–Opitz syndrome (*n* = 3), lysinuric protein intolerance (*n* = 3), propionic acidemia (*n* = 3), adenylosuccinate lyase deficiency (*n* = 3), methylmalonic aciduria type cblB (*n* = 2), long-chain-3-hydroxyacyl CoA dehydrogenase deficiency (*n* = 2), citrullinemia type I (*n* = 2), very-long-chain-acyl-CoA dehydrogenase deficiency (*n* = 2), Mevalonic aciduria (*n* = 2), metachromatic leukodystrophy (*n* = 2), Ornithine transcarbamylase deficiency (*n* = 2), Maple syrup urine disease (*n* = 2), molybdenum cofactor deficiency (*n* = 2), TBCK deficiency (*n* = 2), hyperprolinemia, type I (*n* = 2), tyrosinemia I (*n* = 2), argininosuccinic aciduria (*n* = 2), cerebrotendinous xanthomatosis (*n* = 2), beta-mannosidose (*n* = 2), phosphoserine aminotransferase deficiency (*n* = 2), alpha-methylacyl-CoA racemase deficiency (*n* = 2), glutaric aciduria II (*n* = 1), beta-ketothiolase deficiency (*n* = 1), 3-methylcrotonyl-coa-carboxylase deficiency (*n* = 1), short-chain acyl-CoA dehydrogenase deficiency (*n* = 1), alpha-mannosidosis (*n* = 1), mucopolysaccharidosis type III (*n* = 1), malonyl-Coa decarboxylase deficiency (*n* = 1), glutamate formiminotransferase deficiency (*n* = 1), congenital disorder of glycosylation type IIc (*n* = 1), thymidine phosphorylase deficiency (*n* = 1), mucopolysaccharidosis type 3a (*n* = 1), pyruvate dehydrogenase phosphatase deficiency (*n* = 1), dihydrofolate reductase deficiency (*n* = 1), Hartnup (*n* = 1), 2-ketoglutarate dehydrogenase complex deficiency (*n* = 1), argininemia (*n* = 1), citrin deficiency (*n* = 1), pyridoxine-dependent epilepsy (*n* = 1), glutaric aciduria I (*n* = 1), TANGO2 deficiency (*n* = 1), Lesch–Nyhan syndrome (*n* = 1), ornithine aminotransferase (*n* = 1), carbamoyl phosphate synthetase deficiency (*n* = 1), galactosemia (*n* = 1), xanthinuria type 1 (*n* = 1), 3-hydroxy-3-methylglutaryl-CoA lyase deficiency (*n* = 1), combined malonic and methylmalonic aciduria (*n* = 1), L-2-hydroxyglutaric aciduria (*n* = 1), alkaptonuria (*n* = 1), mucopolysaccharidosis type 3c (*n* = 1), cerebral creatine deficiency syndrome 2 (*n* = 1), congenital disorder of glycosylation type Iy (*n* = 1), cystathioninuria (*n* = 1) and 3-methyl-crotonyl-glycinuria (*n* = 1). Here, 61 out of these 122 patient samples received treatment when their samples were acquired. In agreement with national legislation and institutional guidelines, all patients or their guardians approved the possible de-identified use of the remainder of their samples for method validation and research purposes. Most of the samples were measured as a (technical) triplicate, but only the first sample of the triplicate was used in this study.

Feature levels were expressed as a Z-score by subtracting the mean and dividing by the standard deviations, while also including the known IEM patients. The latter is important to prevent artificial clustering of the control and the patient groups. To reduce the contributions of outlier samples in an unbiased fashion, we randomly selected 50% of the data, removed outliers (|Z-score| > 3), and then determined the mean and standard deviation of the resulting samples. This was repeated 100 times and the final population mean and standard deviation were taken to be the average of the 100 means and 100 standard deviations.

Since we did not wish a single or few extreme Z-scores to dominate/affect the findings, we used a truncated version of the Z-scores using the following equation:

(A1) $$\begin{array}{c} \tilde{Z}=\mathrm{sign}\left(Z\right)\left({\alpha \left(Z\right)\;|Z|}^{0.75}+\left[1-\alpha (Z)\right]\left|Z\right|\right)\\ \alpha \left(Z\right)=\frac{1}{1+\exp\left(2-|Z|\right)} \end{array}$$

This transform behaves approximately linear for the region 0 < |Z| < 2 but scales down |Z-scores| when |Z| ≫ 2.

### Appendix A.2. Miller Dataset

We used the metabolomics dataset that was published by Miller et al. [1]. This dataset is available via https://www.ncbi.nlm.nih.gov/pmc/articles/PMC4626538/ (accessed on 1 May 2022). No adjustments were made to the Z-scores. In total, 120 known IEM patient samples and 70 normal samples were included in this dataset. The dataset contains 1203 features, but we chose to only include the 661 annotated features.

### Appendix A.3. Radboudumc Dataset

High-resolution untargeted metabolomics data from blood plasma was measured for 12 separate batches at the Translational Metabolic Laboratory, Radboudumc, The Netherlands using the protocol described by Coene et al. [2]. All patients and control subjects (or their guardians) included in these batches registered their informed consent for the possible use of their left-over body fluid samples from clinical diagnostics for laboratory method validation purposes in their electronic patient records. The study was conducted in accordance with the Declaration of Helsinki, and following national (Dutch WMO (Medical Research Involving Human Subjects Act), article 7:467) and institutional legislation (CMO Radboudumc Nijmegen) on the use of left-over material from clinical diagnostics.

These 12 independent batches were then merged into a single dataset. Next, peak detection and retention time alignment were performed using the XCMS R package [33]. Correction for intensity drift and batch effects was performed using batchCorr R package v0.2.4 [36]. Only positive ion mode data were used for this study. The (merged) dataset contains 41 samples from either patients with a known IEM or abnormal metabolomics profiles. The following IEM were included: Ataxia–Telangiectasia (*n* = 14), glutamate oxaloacetate transaminase deficiency (*n* = 7), S-Adenosylhomocysteine hydrolase deficiency (*n* = 4), AICA-ribosiduria (*n* = 3), N-acetylneuraminic acid synthase deficiency (*n* = 3), argininosuccinic aciduria (*n* = 2), methionine adenosyltransferase I/III (*n* = 1), long-chain 3-ketoacyl-CoA thiolase deficiency (*n* = 1), 5-oxoprolinase deficiency (*n* = 1), cerebrotendinous xanthomatosis (*n* = 1) and very-long-chain acyl-CoA dehydrogenase deficiency (*n* = 1). Three samples had an abnormal metabolite profile. Seven patient samples with Ataxia–Telangiectasia were acquired before starting treatment. Most IEM patient samples were acquired while receiving treatment. Then, 123 samples were assigned to ‘normal’, as they did not receive a diagnosis or had a diagnosis that was non-IEM related. The third group of samples consisted of 510 samples assigned the label ‘undiagnosed patients’. This group included samples measured during screening but where no diagnosis had been established. The dataset contains 6362 features. Feature abundancies were log-transformed and Z-scores were determined in the same manner as described in Section *Erasmus MC dataset*. Note that the ‘undiagnosed patients’ group was also used for determining Z-scores since the average and standard deviation were calculated using all samples in the dataset. The Z-score truncation (Equation (A1)) was not applied to this dataset.

## Appendix B. Detailed Description of Outlier Detection Methods

For this study, we implemented 30 outlier detection methods. A summary of their characteristics can be found in Table A1. Details about their implementations and the used settings for each method are described in this section.

### Appendix B.1. Autoencoder

The TensorFlow with Keras framework (version 2.6.0) was used to create an autoencoder (AE), which consisted of five hidden layers [37,38]. Two hidden layers were used as encoder, having M/2 and M/4 nodes, respectively, where *M* is the number of (input) features. The bottleneck layer consisted of M/10 nodes, and the decoder part consisted of M/4 and M/2 nodes, respectively. For all hidden layers, we used the hyperbolic tangent activation. The output layer had no activation function. The mean squared error between the input and reconstructed profile was used as a loss to train the autoencoder. To prevent overfitting, we used a dropout layer between all layers, with a dropout rate of 0.1. Furthermore, we added uncorrelated normal noise N (μ = 0, σ) to the profiles during batch training, while drawing σ from a uniform distribution U(0.05, 0.1). We used the Adam() optimizer with a learning rate of 0.0001.

Outlier scores were obtained using the mean squared error between the input and the reconstructed profile. For the Erasmus MC dataset, the trained AE (i.e., trained on the train set) was also used to impute the missing values. The same trained AE was also used to calculate the outlier scores for the *AE reconstruction error* method.

### Appendix B.2. ANN Classifier

A simple artificial neural network was trained to distinguish artificial outlier profiles from normal profiles included in the train set. These outlier profiles originated from two types of noise:

1. Uncorrelated normal noise: outlier profiles were drawn from N(μ=0, σ) with three different values for σ, namely 0.25, 0.5, and 1.
2. Subspace perturbation: following the method described by [16], where outlier profiles were acquired by perturbing normal profiles as follows:
  (A2) $$\begin{array}{c} z∼N(0,I\sigma )\\ x_{i}^{\mathrm{negative}}=x_{i}^{\mathrm{positive}}+M\circ z \end{array}$$

where $x_{i}^{\mathrm{positive}}$ is the metabolomics profile (vector) of sample *i*. The vector M contains binary elements with a probability *p* and 1−p of being ‘1’ or ‘0’, respectively. In this study, *p* was set to p=0.3. z is a vector where each element is normally distributed. σ takes different values: 0.25, 0.5, and 1.

Three hidden layers were used with M/2, M/4, and M/10 nodes, respectively, with *M* being the dimensionality of the input. Two settings were explored for the first hidden layer either with or without a bias term. Hyperbolic tangent activation was used for all hidden layers. A single classifier node with sigmoid activation was used for classification. We used binary cross entropy as the loss for training the network with noise and normal profiles being classified as ‘1’ and ‘0’, respectively. Dropout layers were used to prevent overfitting with a dropout rate of 0.1. We use the Adam()optimizer with a learning rate of 0.0001 for training. The output value of the classifier node was used as an outlier score for each sample. Outlier scores were determined after several training steps, namely: 100, 500, 1000, 2000, 5000, 10,000, and $N_{max}$, with the latter being a variable number for each dataset.

### Appendix B.3. ALOCC

We implemented the *ALOCC* method as described by Sabokrou et al., where we used the same AE architecture design as described in Autoencoder [24]. The parameter λ was set to 0.4 such that the reconstruction loss weighted less than the generator loss. Input profiles were corrupted by adding normal noise from N(μ = 0, σ = 0.1). The discriminator network consisted of four hidden layers having M/2, M/4, M/10, and 2 nodes, respectively, with M being the number of features of the input. A single output node with sigmoid activation was used to discriminate between fake (0) and real input samples (1). Dropout layers with a dropout rate of 0.1 were used between all layers in both the generator and discriminator except for the last hidden layer and the output node of the discriminator. Additionally, we used one-sided label smoothing to improve network training [34]. Here, the labels for the real samples are set to 0.9 instead of 1, which prevents the discriminator from being overconfident. We used the Adam() optimizer with a learning rate of 0.0001.

Outlier scores were obtained in two ways: (1) by passing the reconstructed sample (from generator) to the discriminator and (2) by passing the sample directly through the discriminator. The output of the discriminator equals the outlier score, where values closer to 0 suggest stronger abnormality and vice versa. Outlier scores were determined after several training steps, namely: 100, 500, 1000, 2000, 5000, 10,000, and $N_{max}$, with the latter being a variable number for each dataset.

### Appendix B.4. ALAD

We implemented the *ALAD* method as described by Zenati et al. [27]. The encoder network consisted of two hidden layers having M/2 and M/4 nodes, respectively. The decoder network consisted of two hidden layers having M/4 and M/2 nodes, respectively. The latent dimension was set to k= 2, 3, and 4. The two discriminator networks, called $D_{xx}$ and $D_{xz}$, were identical, each having two hidden layers with M/4, M/2, and 2 nodes, respectively. The discriminator network $D_{zz}$ consisted of two hidden layers, with 10k and 5k nodes, respectively. For all discriminator networks, a single node with sigmoid activations was used for classification. For all hidden layers, the hyperbolic tangent activation was used, and dropout was used between all layers (dropout rate = 0.1). We used the Adam() optimizer with a learning rate of 0.0001 for training. Outlier scores were determined by using the mean absolute error between the activations of the last hidden layer in the $D_{xx}$ network between the real and reconstructed profile. Outlier scores were determined after several training steps, namely: 100, 500, 1000, 2000, 5000, 10,000, and $N_{max}$, with the latter being a variable number for each dataset.

### Appendix B.5. AnoGAN

We implemented the *AnoGAN* method as described by Schlegt et al., except for some minor differences [25]. The generator consisted of three hidden layers with M/10, M/4, and M/2 nodes, respectively, where *M* is the dimension of the output (i.e., total number of features of the metabolomics profiles). *k*-Dimensional uncorrelated normal noise N(μ = 0, σ = 1) was used as input for the generator, where *k* was set to 2 and 3. The discriminator consisted of three hidden layers with M/2, M/4, and 2 nodes, respectively, with a classifier node (sigmoid activation) as output. In both networks, we used dropout layers between all hidden layers (dropout rate = 0.1), except between the latent input space and the first hidden layer in the generator, and between the last hidden layer and the output node of the discriminator. One-sided label smoothing was used to improve network training (see *ALOCC*). We used the Adam() optimizer with a learning rate of 0.0001 for training.

Outlier scores were obtained by first approximating a point in the latent space of the generator that outputs a sample that more closely resembles the ‘query sample’ (i.e., sample for which the outlier scores is determined). This is done by considering the following loss:

(A3) $$\begin{array}{c} L=\sum |x-G\left(z_{\gamma }\right)|+\sum |f\left(x\right)-f\left(G\left(z_{\gamma }\right)\right)| \end{array}$$

with *G* indicating the generator and f indicating the activations of the second hidden layer in the discriminator. $z_{\gamma }$ indicates the point in the latent space that more closely resembles the query sample x. Note, that the weights for the generator and discriminator are kept fixed when backpropagation was used to approximate $z_{\gamma }$. Per (query) sample, we used 50 backpropagation steps, using the Adam() optimizer with a learning rate of 0.005. Outlier scores were determined after several training steps, namely: 100, 500, 1000, 2000, 5000, 10,000, and $N_{max}$, with the latter being a variable number for each dataset.

### Appendix B.6. ABOD

*ABOD* from the pyOD toolkit was applied to each dataset and is available at https://github.com/yzhao062/Pyod (accessed on 1 May 2022) [32,39].

#### Appendix Average of Top 10 Most Extreme Absolute Z-Scores

For each sample, we determined the top 10 most extreme absolute Z-scores. The average of these 10 |Z-scores| was used to obtain the outlier score.

### Appendix B.7. COPOD

*COPOD* from the pyOD toolkit was applied to each dataset [40]. Since *COPOD* has no learning parameters obtained from the train set, we ran a leave-x-out procedure for the evaluation/test set in order to obtain outlier scores. For clarity, we took *n* samples from the IEM patient samples and *n* samples from the normal samples, which originated from the evaluation/test set. These 2n samples were concatenated with the train set, and *COPOD* was applied to obtain the outlier scores for these samples. This procedure was repeated until all samples in the evaluation/test set had an outlier score. The size of the concatenated data was always equal to 2n. For the Erasmus MC dataset, n=5; for the Miller dataset, n=2; and for the Radboudumc dataset, n=5.

### Appendix B.8. COF

*COF* from the pyOD toolkit was applied to each dataset [41]. The following settings were used: n_neighbors= *N*, method=’fast’, with *N* being 10% and 5% of the number of total samples in the train set. Since COF had no learning parameters obtained from the train set, we ran a leave-x-out procedure for the evaluation/test set in order to obtain outlier scores (see *COPOD*).

### Appendix B.9. DeepSVDD

*DeepSVDD* from the pyOD toolkit was applied to each dataset [23]. The following settings were used: use_ae=True, random_state=1, hidden_activation= ’tanh’, dropout_rate=0.1, preprocessing=False, and hidden_neurons = [M/2, M/4], where *M* is the dimension of the input. For the parameter epochs, we used 10, 50, 100, 500, and 1000.

### Appendix B.10. ECOD

*ECOD* from the pyOD toolkit was applied to each dataset [42]. Since *ECOD* has no learning parameters obtained from the train set, we ran a leave-x-out procedure for the evaluation/test set in order to obtain outlier scores (see *COPOD*).

### Appendix B.11. HBOS

*HBOS* from the pyOD toolkit was applied to each dataset [43]. For the parameter n_bins, we used five different settings, namely, 50, 100, 250, 500, and 1000. For the parameter alpha, we choose 0.1 and 0.5.

### Appendix B.12. Isolation Forest

The *Isolation Forest* algorithm, as implemented by Scikit-learn (version 0.23.3), was used to determine outlier scores [21]. Three different settings were used for n_estimators, namely 500, 1000, and 2000. The function score_samples() from Scikit-learn was used to obtain the outlier scores.

### Appendix B.13. KDE

Kernel density estimation (*KDE*) was performed using the KernelDensity() function from Scikit-learn. We used the ’gaussian’ setting for the parameter kernel. Five different settings were used for bandwidth, namely 0.05, 0.1, 0.25, 0.5, and 0.75. We used the score_samples() function to obtain the outlier scores for this method.

### Appendix B.14. Feature Bagging

Feature bagging (FB) from the pyOD toolkit was applied to each dataset [44]. *LOF* was used for outlier detector methods.

### Appendix B.15. KNN

K-nearest neighbor (*KNN*) from the pyOD toolkit was applied to each dataset. The following settings were used: n_neighbors=*N*, with *N* being 5%, 10%, and 25% of the number of samples in the train set. For the setting metric, we used ’euclidean’, ’l1’, and ’l2’. For the setting method, we used ’largest’, ’mean’, and ’median’.

### Appendix B.16. LMDD

*LMDD* from the pyOD toolkit was applied to each dataset [45]. The following settings were used: n_iter=50, dis_measure=’aad’, random_state=None. Since *LMDD* has no learning parameters obtained from the train set, we ran a leave-x-out procedure for the test set in order to obtain outlier scores (see *COPOD*).

### Appendix B.17. LOCI

*LOCI* from the pyOD toolkit was applied to each dataset [46]. For the setting alpha, we used the values 0.1, 0.25, and 0.5. For the setting k, we used 1, 2, and 3.

### Appendix B.18. LODA

*LODA* from the pyOD toolkit was applied to each dataset [47]. For the setting n_random_cuts, we used the values 100, 250, and 500. For the setting n_bins, we used ’auto’, 10, 50, 100, and 250.

### Appendix B.19. LOF

The local outlier factor (*LOF*) algorithm from Scikit-learn was trained on each train set [15]. We used the following settings for the parameters: n_neighbors= *N*, novelty=True, with *N* being 5%, 10%, and 25% of the number of samples in the train set. We used the score_samples() function to obtain the outlier scores for this method.

### Appendix B.20. LUNAR

The implementation of learnable unified neighborhood-based anomaly ranking (*LUNAR*) can be found at https://github.com/mbongaerts/pyod/tree/LUNAR/pyod [16] (accessed on 28 June 2022). We used the following settings for the parameters: n_neighbors=*N*, negative_sampling= ’SUBSPACE’, test_size=0.05, lr=0.0001, scaler=None, n_epochs=2000, with *N* being 5%, 10%, and 25% of the number of samples in the train set. Note that *LUNAR* uses an internal cross-validation approach on the train set to determine the optimal network weights.

### Appendix B.21. Mahalanobis

In line with the proposed method, as described Brini et al. [14], we estimated the covariance matrix for each dataset using the ShrunkCovariance method from Scikit-learn. The Mahalanobis distance obtained from the estimated covariance matrix was used as the outlier score.

### Appendix B.22. MO-GAAL

*MO-GAAL* from the pyOD toolkit was applied to each dataset [26]. The following settings were used: lr_d=0.0001, lr_g=0.0001, stop_epochs=5000, decay=1e-06, momentum=0.9. For the parameter k we used the values 1 and 5.

### Appendix B.23. OC-SVM

The one-class support vector machine (*OC-SVM*) algorithm from Scikit-learn was trained on each train set [17]. We used the following settings for the parameters: gamma = ’auto’. For the parameter kernel, we used ’rbf’ and ’linear’. We used the score_samples() function to obtain the outlier scores for each sample in the test set.

### Appendix B.24. OC-CNN

We implemented the one-class convolutional neural network (*OC-CNN*) method as described by Oza et al. with some additional changes [22]. Instead of using a convolutional neural network as the feature extractor, we used the latent space from the trained AE (see *Autoencoder*) as input for the classifier. The classifier consisted of two hidden layers with *M* and M/2 nodes, respectively, where M is the dimension of the input (i.e., the dimension of the AE latent space). Two settings were explored for the first hidden layer either with or without a bias term. Hyperbolic tangent activation was used for the hidden layers and a single node with sigmoid activation was used for classification. As an artificial outlier class, three types of noise were explored: (1) uncorrelated normal noise N(μ = 0, σ), (2) noise from a uniform distribution U(-σ, σ), and 3) subspace perturbation (see *ANN classifier*). Three different values for σ were used: 0.1, 0.5, and 1. Outlier scores were determined after several training steps, namely: 100, 500, 1000, 2000, 5000, 10,000, and N${}_{max}$, with the latter being a variable number for each dataset. Dropout layers were used to prevent overfitting with a dropout rate of 0.1. We used the Adam() optimizer with a learning rate of 0.0001.

### Appendix B.25. PCA reconstruction error

Following the procedure described by Engel et al., we calculated the Q-values by performing principle component analysis using *p* components [13]. Outlier scores were obtained by projecting each of the first *p* principle components (as determined from the train set), where each sample is reconstructed from its lower dimensional representation, using the inverse transformation. The Q-value, i.e., outlier score for each sample was calculated from the following equation:

(A4) $$Q\text{-}\mathrm{value}=||x-\tilde{x}{\left|\right|}^{2}$$

where *x* is the input sample and $\tilde{x}$ is the reconstructed sample. Parameter *p* was set to 5, 10, 25, 50, 75, and 100.

### Appendix B.26. R-graph

*R-graph* was developed by You et al. [20]. In this study, we used the function elastic_net_subspace_clustering() obtained from https://github.com/ChongYou/subspace-clustering (accessed on 3 May 2022) to determine the self-representation of each dataset. The following parameters were used: gamma_nz=True, tau=1.0, algorithm= ’lasso_cd’. Parameter gamma was set to 50 and 100. Parameter n_nonzero=N, with N being 5%, 10%, and 25% of the total number of samples in the train set. We used 10, 25, 50, and 100 transition steps in the Markov process to obtain the final (average) outlier scores for each sample. Since *R-graph* has no learning parameters that are obtained from the train set, we ran a leave-x-out procedure for the evaluation/test set in order to obtain outlier scores (see *COPOD*).

### Appendix B.27. SVDD

The support vector data description (*SVDD*) method was implemented in Python and obtained from the GitHub repository https://github.com/iqiukp/SVDD-Python (accessed on 31 March 2022) [18]. The model was trained without using outlier samples. The following settings were used: gamma=’auto’. For parameter kernel, we used ’rbf’ and ’linear’. Parameter C was set to 0.1, 0.5, 1, and 2.

### Appendix B.28. SOS

*SOS* from the pyOD toolkit was applied to each dataset [19]. The following settings were used: metric= ’euclidean’, eps=1e-05, perplexity= *N*, with *N* being 5%, 10%, 25%, and 50% of the number of samples in the train set. Since *SOS* has no learning parameters obtained from the train set, we ran a leave-x-out procedure for the evaluation/test set to obtain outlier scores (see *COPOD*).

### Appendix B.29. SOD

*SOD* from the pyOD toolkit was applied to each dataset [48]. The following settings were used: n_neighbors= *N*, ref_set= 0.5N, alpha= 0.8, with *N* being 5%, 10%, and 25% of the number of total samples in the train set, respectively. Since *SOD* has no learning parameters obtained from the train set, we ran a leave-x-out procedure for the evaluation/test set in order to obtain outlier scores (see *COPOD*).

**Table A1:** Overview of outlier detection methods used in this study.

Nr.	Name Method	Type	Summary of Working Principle	Distance Metric	Performs (Indirect) Dimensionality Reduction	Uses a Form of an (Artificial) Outlier Class for Training
1	AE reconstruction error	ANN	Reconstruction error between autoencoder reconstructed sample and input sample	l2	Yes, latent space of the AE	
2	ALOCC	ANN (GAN)	GAN-based method, but replaces generator for an autoencoder. Outlier scores are obtained from discriminator that discriminates reconstructed samples from real samples.		Yes, latent space of the generator	
3	ALAD	ANN (GAN)	GAN with multiple generator and discriminator networks. Outlier scores obtained from the l1 error between the activations in a hidden layer of the discriminator between reconstructed and input sample.	l1	Yes, latent space of the generator	Generated samples from the generator
4	AnoGAN	ANN (GAN)	Reconstruction error between reconstructed and input sample after finding an approximate point in latent space of a query sample.	l1	Yes, latent space of the generator	Generated samples from the generator
5	ANN classifier	ANN	ANN classifier that distinguishes noise from normal samples.		Yes	Random noise or subspace perturbation
6	ABOD	Probabilistic	A comparison of the variance of angles between query sample and other samples in the dataset. Outlier samples are expected to have a lower (angle) variance.		No	
7	Average of top 10 most extreme absolute Z-scores	Probabilistic				
8	COPOD	Probabilistic	Combines empirical cumulative distributions in a copula to estimate a ‘probability’ per sample.		No	
9	COF		Compares the average chaining distance of a point with the average chaining distance of the k-nearest neighbors.		No	
10	DeepSVDD	Support vector + ANN	Minimum volume of sphere containing majority of the normal samples using an ANN for non-linear mapping.		Yes	
11	ECOD		Computes left- and right-tail univariate empirical cumulative distribution functions (ECDFs) per feature. ECOD uses the uni-variate ECDFs to estimate tail probabilities for the datapoint and aggregates these tail probabilities to a final outlier score.		No	
12	HBOS	Density / proximity	Builds a histogram for each dimension and aggregates the results from each histogram into a single outlier score.			
13	Isolation Forest	Ensemble	Number of splits needed to isolate a sample.		No	
14	KDE	Density / proximity / Probabilistic	Density based on Gaussian kernel density approximation.	Depends on the used kernel	No	
15	Feature bagging	Ensemble	Ensemble of detectors that use a random subset of features.			
16	KNN	Density / proximity	Mean, largest, or median distance of the k-nearest neighbors.	Euclidean, l1, l2	No	
17	LMDD					
18	LOCI	Density / proximity	Compares the density of a sample with the density of its neighborhood. Density is measured by considering the multi-granularity deviation factor.			
19	LODA	Ensemble	Using an ensemble of one-dimensional histograms by projecting the data to a (random) one-dimensional space.		Inconclusive	
20	LOF	Density / proximity	Compares the average distance of a sample to its neighboring samples with the average distance of those samples with their neighborhood.		No	
21	LUNAR	Graph + ANN	Uses a graph neural network (GNN), where the graph is determined from the local neighborhood of each sample. GNN is trained using an artificial outlier class.		Yes, depending on number of nodes in hidden layers	Subspace perturbation
22	Mahalanobis	Density / proximity	Mahalanobis calculated from the estimated covariance matrix.	Mahalanobis	No	
23	MO-GAAL	ANN (GAN)	GAN with multiple generators to generate different parts of the normal data.			
24	OC-SVM	Support vector	Finds a hyperplane that maximizes the distance of the normal data to the origin.		No	Origin is outlier class
25	OC-CNN	ANN	ANN classifier that distinguishes noise from normal samples after feature extractor		Yes, latent space of the feature extractor (AE)	Random noise or subspace perturbation
26	PCA reconstruction error	Density / proximity	Reconstruction error between input sample and reconstructed sample after projection to lower dimensional space from PCA.	l2	Yes, PCA	
27	R-graph	Graph	Represents each samples as a linear combination of other samples, using a Markov process to propagate scores though the graph.		Projection in a (lower) subspace	
28	SVDD	Support vector	Minimum volume of sphere containing majority of the normal samples (using a kernel).			
29	SOS	Graph		Euclidean		
30	SOD		Each sample’s outlierness is evaluated in a relevant subspace.		Projection in a (lower) subspace	

## Appendix C. Optimal Settings for Each Dataset and Outlier Detection Method

**Table A2:** Optimal settings for each method and dataset based on the highest average AUC across the evaluation CVs.

Method	Setting	Erasmus MC	Miller	Radboudumc
*ALAD*	epochs	100	10,000	500
*ALAD*	latent_dim	4	3	4
*ALOCC* D(x)	epochs	100	5000	100
*ALOCC* G(D(x))	epochs	2000	500	1000
*ANN*	bias	False	False	True
*ANN*	epochs	500	2000	5000
*ANN*	noise	normal	subspace_ perturbation	normal
*ANN*	std	1	1	0.25
*AnoGAN*	epochs	2000	100	2000
*AnoGAN*	latent_dim	3	3	3
*DeepSVDD*	epochs	10	1000	1000
*HBOS*	alpha	0.1	0.1	0.5
*HBOS*	n_bins	100	50	250
*Isolation Forest*	n_estimators	500	500	500
*KDE*	bandwidth	0.25	0.25	0.5
*KNN*	distance	euclidean	euclidean	l1
*KNN*	method	mean	mean	mean
*KNN*	n_neighbors_frac	0.05	0.05	0.1
*LOCI*	alpha	0.1	0.5	0.5
*LOCI*	k	1	1	2
*LODA*	n_bins	50	10	auto
*LODA*	n_random_cuts	500	500	100
*LOF*	n_neighbors_frac	0.05	0.1	0.05
*LUNAR*	n_neighbors_frac	0.1	0.1	0.1
*MO-GAAL*	k	5	5	5
*OC-CNN*	bias	True	True	True
*OC-CNN*	epochs	5000	15000	5000
*OC-CNN*	noise	uniform	normal	uniform
*OC-CNN*	sigma	0.5	1	0.5
*OC-SVM*	kernel	rbf	rbf	linear
*PCA reconstruction error*	n_components	50	25	100
*R-graph*	gamma	100	100	50
*R-graph*	n_nonzero_frac	0.05	0.25	0.25
*R-graph*	steps	100	25	10
*SOD*	n_neighbors_frac	0.05	0.05	0.25
*SOS*	perplexity_frac	0.5	0.25	0.5
*SVDD*	C	2	1	2
*SVDD*	kernel	rbf	linear	linear

## Appendix D. PCA Prior to Applying the Outlier Detection Method (Erasmus MC)

We trained the outlier detection methods on varying numbers of features by performing principle component analysis (PCA). PCA was performed on the train and test set (including both normal and IEM patient samples). After the transformation, a certain number of principle components was selected and the train and test set were disjointed. Each outlier detection method was trained on the train set, and tested on the test set in the same manner as described in *Methods* but without parameter setting selection. This experiment was performed on the Erasmus MC dataset and for a fixed set of settings for each method (see Table A3).

Figure A1 shows the AUC for each method and a varying number of PCs. Overall, we observe an increasing AUC for an increasing number of PCs. The results indicate that for some methods (e.g., *COPOD* and *LMDD*) an optimal number of PCs may exist. Furthermore, we observe that the influence of the number of PCs largely differs among the methods.

**Table A3:** Settings used for the experiment as described in Appendix B.

Method	Setting	Value
OCSVM	kernel	rbf
*SVDD*	C	1
*SVDD*	kernel	rbf
*PCA reconstruction error*	n_components	9
*ALOCC* D(x)	epochs	10,000
*OC-CNN*	bias	False
*OC-CNN*	epochs	10,000
*OC-CNN*	noise	uniform
*OC-CNN*	sigma	0.1
ANN	bias	False
ANN	epochs	10,000
ANN	noise	normal
ANN	std	0.25
*LODA*	n_bins	50
*LODA*	n_random_cuts	500
*LOCI*	alpha	0.1
*LOCI*	k	1
*R-graph*	gamma	100
*R-graph*	n_nonzero_frac	0.05
*R-graph*	steps	100
*MO-GAAL*	k	5
*HBOS*	alpha	0.1
*HBOS*	n_bins	100
*DeepSVDD*	epochs	10
*SOS*	perplexity_frac	0.5
*SOD*	n_neighbors_frac	0.1
*Isolation Forest*	n_estimators	500
*LOF*	n_neighbors_frac	0.05
*KNN*	distance	euclidean
*KNN*	method	largest
*KNN*	n_neighbors_frac	0.05
*KDE*	bandwidth	0.25
*LUNAR*	n_neighbors_frac	0.1
*AnoGAN*	epochs	10,000
*AnoGAN*	latent_dim	3
*ALAD*	epochs	10,000
*ALAD*	latent_dim	3

## Appendix E. PCA Prior to Applying the Outlier Detection Method for Radboudumc Dataset

A subset of outlier detection methods was applied on the PCA transformed Radboudumc dataset, where we included various numbers of principle components (PCs). Note that all samples in the dataset were used to perform this transformation, which includes the 510 undiagnosed patient samples, the 38 IEM patient samples, the three abnormal samples, and the 123 normal samples. AUCs were obtained in the same manner as described in Methods. Figure A2 shows the effect of an increasing number of PCs on the (average) AUC for each investigated method. Interestingly, we observe that for *PCA reconstruction error*(with 10 PCs) that we obtained an AUC of 0.84, which is higher than the AUC obtained when *PCA reconstruction error* is applied directly to the dataset (see Figure 2). Similarly, we observe good AUC (≥0.78) for *ABOD*, *LOF*, *KDE* when 10/20 PCs are used while AUCs are low (AUC ≈ 0.58–0.66) when applied directly to the dataset. These findings support the results that are obtained from a similar experiment performed on the Erasmus MC dataset (Appendix D). Clearly, some methods benefit from an initial PCA transform prior to applying the outlier detection method.

Next, we determined the recall, precision, and balanced accuracy at the ‘closest-to-(0,1)’ point and ‘recall${}_{P}$ = 0.9 point’ as can be observed in Figure A3 and Figure A4.

## Appendix M. Effect of Scaling Based on Different Groups on Outlier Detection Performance

We investigated the effect of the initial Z-score scaling on the IEM detection performance for six outlier detection methods for the Miller dataset. The first Z-score scaling is based on the 70 control samples, where the mean and standard deviation for each metabolite are calculated from these samples. For the second Z-score scaling method, we used all 190 samples to determine this mean and standard deviation (per metabolite) and using the iterative procedure as described in Appendix A. For six outlier detection methods and both scaling methods we calculated the AUC using the 120 IEM patient samples, and 25 control samples as the test set (i.e., 45 control samples were in the train set) (Figure A12). The results indicate that for 5/6 outlier detection methods, the AUC dropped for scaling method 2 with respect to scaling method 1.
